# Human papilloma virus, DNA methylation and microRNA expression in cervical cancer (Review)

**DOI:** 10.3892/or.2014.3142

**Published:** 2014-04-16

**Authors:** HILDA JIMÉNEZ-WENCES, OSCAR PERALTA-ZARAGOZA, GLORIA FERNÁNDEZ-TILAPA

**Affiliations:** 1 Clinical Research Laboratory, Academic Unit of Biological Chemical Sciences, Guerrero Autonomous University, Colonia Haciendita, Chilpancingo, Guerrero 39070, Mexico; 2 Direction of Chronic Infections and Cancer, Research Center for Infectious Diseases, National Institute of Public Health, Cerrada los Pinos y Caminera, Colonia Santa María Ahuacatitlán, Cuernavaca, Morelos 62100, Mexico

**Keywords:** miRNAs, methylation, human papilloma virus, cervical cancer

## Abstract

Cancer is a complex disease caused by genetic and epigenetic abnormalities that affect gene expression. The progression from precursor lesions to invasive cervical cancer is influenced by persistent human papilloma virus (HPV) infection, which induces changes in the host genome and epigenome. Epigenetic alterations, such as aberrant miRNA expression and changes in DNA methylation status, favor the expression of oncogenes and the silencing of tumor-suppressor genes. Given that some miRNA genes can be regulated through epigenetic mechanisms, it has been proposed that alterations in the methylation status of miRNA promoters could be the driving mechanism behind their aberrant expression in cervical cancer. For these reasons, we assessed the relationship among HPV infection, cellular DNA methylation and miRNA expression. We conclude that alterations in the methylation status of protein-coding genes and various miRNA genes are influenced by HPV infection, the viral genotype, the physical state of the viral DNA, and viral oncogenic risk. Furthermore, HPV induces deregulation of miRNA expression, particularly at loci near fragile sites. This deregulation occurs through the E6 and E7 proteins, which target miRNA transcription factors such as p53.

## 1. Introduction

Cervical cancer is one of the most frequently occurring malignant tumors in women worldwide, with ~470,000 new cases and 233,000 deaths per year (1). Squamous cell cervical carcinoma represents approximately 80% of cases. Cervical cancer develops through well-defined pre-malignant lesions, which are known as cervical intraepithelial neoplasia (CIN), ranging from grades I to III (2). Cervical adenocarcinomas represent 10–20% of cases, but the preceding stages are not well characterized (2). The high-risk HPV (HR-HPV), as well as environmental, immunological, genetic and epigenetic factors, are among the etiological causes contributing to cervical carcinogenesis; progression of precursor lesions to invasive cancer is influenced by HR-HPV infection (3,4). Although the mechanisms by which HR-HPV induces changes to the host’s genome and epigenome are still unknown, it has been established that integration of the viral DNA into the cellular genome causes genetic (deletions, amplifications and DNA rearrangements) and epigenetic (modifications to the DNA methylation status and aberrant miRNA expression) alterations. These result in the silencing of tumor-suppressor genes and the overexpression of oncogenes favoring tumor progression (5–8).

Epigenetic modifications are just as important as genetic modifications in terms of regulating gene expression and controlling disease onset. It has been shown that epigenetic silencing of some miRNA genes is functionally involved in cervical carcinogenesis (2,5,7). The interaction between HR-HPV and miRNAs occurs at different times during carcinogenesis, given that: i) some miRNA loci localize to fragile sites, which are the sites where HR-HPV inserts its DNA; ii) proteins encoded by HR-HPV can influence miRNA expression within the host cell and iii) it has been observed that the E6 and E7 proteins of HR-HPV modulate the expression of DNA methyltransferases, which are enzymes that regulate gene expression by methylating promoter regions (2,5,9,10).

Changes in the expression profile of miRNAs have been reported in cervical cancer cell lines, cervical cancer tissue and precursor lesions (1,11–13). Similarly, studies conducted in cell lines suggest that HPV participates in deregulating miRNA expression by modifying the expression profile of miRNAs associated with the presence of HPV and the viral genotype (1,5,11,14,15). Given that a considerable number of miRNAs are subject to epigenetic regulation, it has been proposed that aberrant methylation of miRNA promoters is one of the mechanisms responsible for deregulated miRNA expression in cervical cancer (2,12,16). Here, we analyzed the influence of methylation on miRNA expression as well as on the expression of proteins that regulate cellular processes and participate in carcinogenesis. We further discuss the likelihood of HPV inducing modifications in the methylation status of miRNA promoters in cervical cancer. Lastly, we also assess the relationship between HR-HPV infection, methylation and miRNA expression.

## 2. HPV and cancer

HPV is one of the most common sexually transmitted infections worldwide and is associated with a wide spectrum of benign and malignant neoplasias (17). HPV is the second infectious agent implicated in cancer development, after *Helicobacter pylori* (17). It is estimated that 5.2% of all types of cancer can be attributed to HPV infection; HPV has been associated with 90–93% of anal cancer cases, 12–63% of oropharyngeal cancer cases, 36–40% of penile cancer cases, 40–51% of vulvar cancer cases, 40–64% of vaginal cancer cases and 99.7% of cervical cancer cases (17,18). Approximately 100 HPV subtypes with genetic variations and different oncogenic potentials have been identified and classified into three groups: high-risk (types 16, 18, 31, 33, 35, 39, 45, 51, 52, 56, 58, 59, 68, 73 and 82); probable high-risk (types 26, 53 and 66) and low-risk (types 6, 11, 40, 42, 43, 44, 54, 61, 70, 72, 81 and CP6108) (4,19).

## 3. HPV and cervical cancer

Epidemiological and molecular studies have documented the causal link between HR-HPV infection and cervical cancer (3,18,20). HR-HPV subtypes HPV 16, HPV 18, and HPV 31 have been detected in 99.7% of squamous cell cervical carcinomas and in 94–100% of adenocarcinomas and adenosquamous carcinomas (12). It is estimated that ~11.4% of women worldwide and 9.4% of Mexican women are at risk of contracting an HPV infection at some point during their lives (21). Furthermore, it is estimated that >80% of sexually active women become infected with HPV, and >50% of young women are infected after their first sexual intercourse. Almost 90% of infections are spontaneously eliminated during a 3-year period, with only 10% becoming persistent infections. Among the latter, only 1% develop into cervical cancer (10,21).

Persistent HPV infection is required for normal cells to transform into cancerous cells (21). An important step in malignant progression is the integration of HPV into the host’s genome (9,10,21). It appears that the integration event does not happen randomly. Although almost all chromosomes are susceptible, certain regions of the human genome are favored for viral DNA insertion, such as fragile sites, rupture and translocation points, and transcriptionally active regions (10). HPV integration into the cellular genome has several implications: i) it allows permanent expression of the E6 and E7 oncoproteins, which promote cell transformation and immortalization by inactivating the p53 and Rb tumor-suppressor genes, respectively, as well as other proteins that participate in cell adhesion, apoptosis, cell cycle, DNA repair, cellular metabolism, and signal transduction regulating transcription and translation; ii) viral integration near or within a gene can eventually lead to cell growth and proliferation alterations and iii) viral integration can induce epigenetic modification of viral and cellular genes, which may affect their expression. A series of epigenetic alterations in the cellular and viral genomes can occur during each stage of cervical cancer (7,10,21).

## 4. DNA methylation in cervical cancer

Regulation of gene expression is a vital process that determines the profile of proteins required to ensure the proper occurrence of processes including development, cellular differentiation, organogenesis, cellular stress response and programmed cell death (22). In normal tissues, epigenetic events such as DNA methylation, histone acetylation and expression of miRNAs, and other small RNAs regulate the expression of genes participating in the activation of differentiation processes as well as cellular functions that contribute to cellular homeostasis (23,24). Twenty-five years ago, it was discovered that epigenetic modifications participate in cancer development, leading to uncontrolled cell proliferation (23). One of the most widely studied epigenetic mechanisms is DNA methylation, a reversible reaction catalyzed by DNA methyltransferase (DNMT) enzymes. DNMT1 is a maintenance methyltransferase that preserves the methylation pattern during each cellular division. DNMT3a and DNMT3b are *de novo* methyltransferases (25,26). DNMTs add a methyl group onto carbon 5 of cytosine residues adjacent to guanine residues (5′-CpG-3′), which mainly occurs in CpG islands. CpG islands are generally found in the promoter regions of protein-coding genes, and expression is silenced upon their methylation. Non-coding genes, such as miRNAs, are also susceptible to regulation by methylation (25,26).

Global DNA hypomethylation in repetitive regions and hypermethylation in CpG island regions of tumor-suppressor gene promoters are DNA modifications that are commonly found early during cancer development (3,27). Alterations to the DNA methylation pattern, which have also been described in cervical cancer, contribute to genomic instability, chromosomal rearrangements, and silencing of coding and non-coding genes, such as miRNAs (2,20,28–30). Silencing of tumor-suppressor genes through DNA hypermethylation has been linked to the development of different types of cancers, including cervical cancer, and is frequently associated with poor clinical results (Table I). However, silencing of tumor suppressor miRNAs through hypermethylation of CpG islands in their promoter regions has also been implicated in carcinogenesis (30,31).

## 5. HPV and DNA methylation

It is thought that HR-HPV can induce changes in DNA methylation and histone acetylation and also cause aberrant miRNA expression (6). Little is known concerning the role of oncogenic viruses in the modification of cellular DNA methylation patterns (35). The hepatitis B, hepatitis C, Kaposi’s sarcoma-associated and Epstein-Barr viruses interact with DNMTs, modulating their expression. As a result, viral and cellular genes are trans-activated and trans-repressed, respectively (6,35,36). Although the relationship between HPV and aberrant methylation in cervical cancer is not well understood, some authors have suggested that HPV interferes with the cellular DNA methylation machinery, either to conceal itself or as part of its viral cycle (6,35,37). Some investigators have described that upon HPV 16 infection, cellular DNA undergoes epigenetic alterations induced by the E6 and E7 oncoproteins (35,38,39). It has been proposed that methylation has arisen as a defense mechanism by the host cell to silence viral DNA (6,40,41).

**Figure 1:**
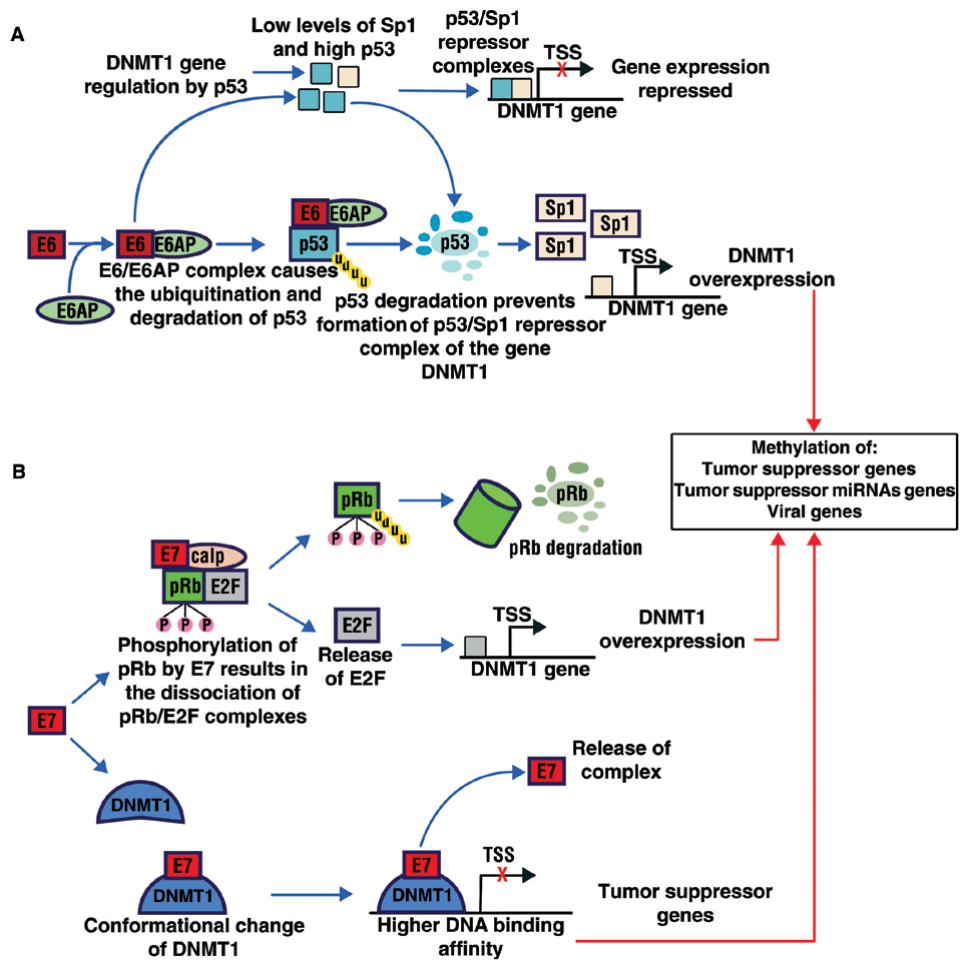
Regulation of viral and cellular gene methylation by the E6 and E7 oncoproteins of HR-HPV. Integration of viral DNA into the cellular genome causes genetic and epigenetic alterations. The E6 and E7 oncoproteins of HR-HPV increase the expression and activity of DNA methyltransferases, particularly DNMT1. (A) Binding of p53 to Sp1 (p53/Sp1) forms a repressor complex for DNMT1 transcription. Degradation of p53 by E6 avoids the formation of this repressor complex and Sp1 induces the expression and activity of DNMT1. (B) E2F positively regulates the promoter activity of DNMT1. Binding of E7 to pRb (E7/pRb) causes the release of E2F, favoring the expression of DNMT1. Binding of E7 to DNMT1 (E7/DNMT1) induces a conformational change in DNMT1, exposing its DNA binding site and promoting DNA binding; once the E7/DNMT1 complex binds DNA, DNMT1 closes on the DNA and maintains a stable DNMT1/DNA interaction, and E7 dissociates from the complex. Overexpression of DNMT1 results in hypermethylation of tumor-suppressor gene promoters, which leads to cellular transformation and tumorigenesis. HR-HPV, high-risk HPV; DNMT, DNA methyltransferase.

The E6 and E7 oncoproteins of HR-HPV increase the expression and activity of DNMT1 (39). E6 does so by degrading p53 (35,39) (Fig. 1A). In the cervical cancer cell lines SiHa and CaSki, knockdown of E6 is associated with an increase in p53 and a decrease in DNMT1 expression (35,39). In contrast, Lin *et al* (42) showed that p53 negatively regulates DNMT1 expression both in cell lines and in lung cancer patients. p53 binds to the specificity protein 1 (Sp1) and chromatin-remodeling proteins, and this complex then binds to the promoter region of DNMT1. The formation of the complex inhibits Sp1 from activating the transcription of DNMT1 (42). Normally, Sp1 induces degradation of p53 by MDM2-mediated ubiquitination (Sp1/p53/MDM2 complex) and induces overexpression of DNMT1 (42). Non-small cell lung cancer patients with mutations in p53 and patients with alterations in p53 and/or Sp1 showed hypermethylated promoter regions of tumor-suppressor genes (p=0.003–0.016), most likely due to DNMT1 overexpression (42).

Modulation of DNMT1 expression by E7 (Fig. 1B) can occur in two different ways: i) indirectly, through E7 binding to pRb, which releases the transcription factor E2F; given that conserved E2F-binding sequences exist at the transcription start site for DNMT1, the release of E2F results in the regulation of the DNMT1 promoter activity and ii) directly, by binding of E7 to DNMT1 (38). It has been proposed that the E7/DNMT1 complex induces a conformational change in DNMT1, exposing its active site, promoting DNMT1/DNA binding, and binding to S-adenosyl-L-methionine (AdoMet) (38). Once the E7/DNMT1/DNA complex forms, E7 dissociates from the complex, and DNMT1 closes on the DNA, maintaining a stable DNMT1/DNA interaction (35,40). The increase in DNMT1 activity causes aberrant methylation of the cellular genome, resulting in the silencing of tumor-suppressor genes and favoring cellular transformation (38,40).

The role of DNMT1 in cervical carcinogenesis has been reported by Jin-Tao *et al* (43), who used *in vitro* and *in vivo* studies and found that low levels of serum folate and high expression of DNMT1 protein or mRNA were significantly associated with cervical carcinogenesis (p=0.001). Integration of HR-HPV DNA into the host’s genome is an essential step in cervical carcinogenesis, and changes to viral DNA methylation are associated with the oncogenic capacity of HPV (28,29). After its integration into the human genome, the DNA of HPV 16 and HPV 18 is methylated (28,45). However, there are still controversial results regarding the participation of HPV in the aberrant DNA methylation that has been observed in cervical cancer.

Henken *et al* (28) used primary human foreskin keratinocytes (PHFKs) transfected with HPV 16 and HPV 18 to decipher the most important events in HR-HPV-mediated transformation. The authors used a longitudinal *in vitro* system utilizing serial passages and found that the transfected keratinocytes gradually developed dysplastic characteristics that were similar to pre-malignant cervical lesions. After immortalization, only the keratinocytes with HPV DNA integrated into their genome accumulated changes in the methylation patterns in the promoter regions of different genes. After transfecting PHFKs with episomal forms of both HPV 16 and HPV 18, Leonard *et al* (35) and Henken *et al* (28) observed overexpression of DNMT1 and DNMT3B. They also found changes in the methylation status of different cellular genes that were previously reported to be methylated in cervical cancer, most likely due to an early epigenetic reprogramming induced by HR-HPV. Furthermore, Leonard *et al* (35) observed differences in the expression topography of DNMTs in relation to the viral genotype. Following transfection with HPV 16, nuclear expression of DNMT1 was restricted to cells in the basal and early differentiation layers and was decreased in more differentiated cells. However, in cells transfected with HPV 18, nuclear DNMT1 expression was observed in the basal and suprabasal layers as well as in the stratum granulosum, with some cells displaying intense DNMT1 staining. Changes in the expression topography of DNMT3B were similar to those observed for DNMT1. However, the global staining intensity was weaker. Moreover, Leonard *et al* (35) analyzed the whole-genome methylation profile after transfection with the episomal forms of HPV 16 and HPV 18 and found a significant increase in the methylation status of 5,607 and 2,387 genes, respectively. They also found a decrease in the methylation status of 3,568 and 4,160 genes, respectively. Non-overlapping increases and decreases in methylation were found for 2,295 and 1,023 genes, respectively. It is possible that the altered miRNA expression observed in cervical cancer is related to the aberrant methylation of miRNA promoters. Thus, HR-HPV could indirectly induce aberrant miRNA methylation (2,20,46).

## 6. miRNAs and their deregulation in cancer

miRNAs are small, non-coding RNA molecules ~22–25 nucleotides (nt) in size. They are usually phylogenetically conserved with a tissue- and time-specific expression pattern (47,48). miRNAs have been recognized as epigenetic regulators, controlling gene expression without altering the DNA sequence (49). The expression profile of miRNAs in cell lines and cervical cancer tissues suggests that aberrant miRNA expression contributes to the development of cervical cancer and HR-HPV-induced precursor lesions (1,7,50). Defects in miRNA expression have been associated with: i) genetic alterations, such as deletions, amplifications and point mutations and ii) epigenetic alterations, such as histone modifications and aberrant DNA methylation (25,46,48,52).

Regulation of miRNA expression is important to maintain cellular homeostasis. However, the molecular mechanisms regulating miRNA gene transcription are not well understood to date (31,54). Currently, it is thought that miRNA biogenesis is regulated at several levels: i) at a transcriptional level, which consists of pri-miRNA transcription by RNA polymerase II and III; ii) at a post-transcriptional level, consisting of miRNA maturation, which involves the processing of pri-miRNA to pre-miRNA, export into the cytoplasm, and incorporation into the RISC complex and iii) at a level of miRNA localization within the genome (23,46).

Regulation of miRNA expression at the transcriptional level is one of the most important steps in their biogenesis, and genome localization influences their transcription (23,54,55). miRNA genes are encoded within the genome as a unit or in groups of 2 to 19 miRNAs and can reside within introns or exons of coding genes or in intergenic regions (1,23,46,55,56). The miRNA genes localized to intergenic regions have their own promoter, which allows them to be independently transcribed. miRNAs localized to intragenic regions (in introns or exons) can be transcribed independently from the gene in which they reside, as long as they have their own promoter, or they can be transcribed together with the host gene (1,23,46,55,56).

Identification of transcriptional start sites and regulatory regions is critical to understand the mechanisms and transcription factors that mediate miRNA expression (56). In general, miRNA expression can be regulated by: i) DNA-binding factors, such as c-myc and p53; ii) specific transcription factors, such as myocyte enhancer factor-2 (MEF2), PU.1 and REST and iii) growth factors, such as platelet-derived growth factor (PDGF) and transforming growth factor β (TGF-β), among others (46). Given that miRNA genes are expressed in a tissue- and time-specific manner and their promoters contain characteristics such as CpG islands, TATA boxes, TFIIB recognition elements, and initiators that are similar to the promoters of protein-coding genes, miRNA expression can also be regulated by epigenetic mechanisms, such as nucleosome remodeling and DNA methylation (23,31–33,46,54,57).

Regulation of miRNA expression at a post-transcriptional level is essential for the specificity and function of certain miRNAs in a tissue- and time-specific context. miRNAs that are clustered in groups are individually expressed independently from the other miRNAs in the group (58). This suggests that miRNAs are individually regulated at a post-transcriptional level (58). Their localization within the genome appears to be an important factor for the regulation or deregulation of certain miRNAs, given that several miRNAs have been mapped to or fragile sites, minimal regions of loss of heterozygosity, amplification, common breakpoint regions and transcriptionally active regions that have been linked to cancer in humans (2,23,46,50,54). In cancer tissues, miRNA expression profiling has revealed that their expression is either increased or decreased compared with healthy tissue. They are differentially expressed in different types of tumor, cell lineages and tumor stages (32,59). miRNAs play an important role in cervical carcinogenesis, from HPV infection to cancer progression (Table II) (50).

## 7. HPV and miRNA promoter methylation in cervical cancer

It is possible that the aberrant methylation of miRNA promoters is responsible for the altered expression of some miRNA genes with tumor-suppressor or oncogenic functions in cancer (Table III). The role of HR-HPV in altering the cellular DNA methylation status is still controversial. However, it is possible that HR-HPV plays a role in the deregulation of miRNA gene methylation in cervical cancer (2,69,70). Although few studies exist regarding DNA methylation in the deregulation of miRNA expression in cancer, it has been proposed that alterations to the methylation status of miRNA genes could explain the deregulation of miRNA expression in cervical cancer (2,69,70).

In patients and cervical cancer cell lines, it has been observed that silencing of tumor-suppressor miRNAs through aberrant promoter methylation favors cervical carcinogenesis (2,20,69,70). It has been proposed that HR-HPV can lead to modifications in the methylation pattern of miRNA promoters (20,69). Leonard *et al* (35) reported that changes to cellular DNA methylation associated with HPV 16 and HPV 18 are not randomly distributed but rather cluster in specific chromosomal regions, such as the HR-HPV integration regions and regions of chromosomal loss and gain. After cervical cancer cell lines (HeLa, SiHa, CaSki and C33A) were subjected to treatment with hypomethylating agents, decreased methylation levels were found for certain miRNAs, which resulted in their increased expression and concomitant decreased expression of their target genes (2,69,70). It is possible that the HR-HPV genotypes are involved in the methylation processes of miRNAs in cervical cancer (20). However, *in vitro* findings suggest that the methylation events take place after cellular immortalization and are not directly related to the presence of HR-HPV (2). It is likely that identifying the methylation status of miRNAs could be useful for the prognosis of precursor lesions and cervical cancer (2).

Analysis of the methylation status of the three loci encoding the mature hsa-miR-124 (hsa-miR-124-1/-2/-3) in cervical cancer cell lines by Wilting *et al* (2) showed that the three promoter regions of hsa-miR-124 were methylated in SiHa and CaSki cells. In contrast, the methylation levels of the three hsa-miR-124 regions, in particular hsa-miR-124-1, were extremely low in HeLa cells compared with those observed in SiHa and CaSki cells. A decrease in methylation levels and overexpression of hsa-miR-124 were observed after SiHa cells were treated with the hypomethylating agent 5-aza-2′-deoxycytidine (5-Aza) (2). Ectopic expression of hsa-miR-124 in SiHa and CaSki cells decreased the proliferation rate and migratory capacity of the cells (2). Yao *et al* (69) proposed that hypermethylation of miR-432, miR-1286, miR-641, miR-1290, miR-1287 and miR-95 may be related to HR-HPV infection. Following treatment with 5-Aza, miRNAs were only overexpressed in cervical cancer cell lines infected with HR-HPV (CaSki, HeLa and SiHa) and not in a cell line without HPV (C33A). Wilting *et al* (70) reported that transcriptional repression of hsa-miR-149, hsa-miR-203, hsa-miR-375 and hsa-miR-638 was associated with an increase in methylation levels of these miRNAs. Treatment of the SiHa cell line with the hypomethylating agent 5-Aza induced an increase in the expression of hsa-miR-149, hsa-miR-203 and hsa-miR-375. However, no increased expression was observed for hsa-miR-638. In accordance with these findings, a decrease in the methylation levels of hsa-miR-149, hsa-miR-203 and hsa-miR-375 but not hsa-miR-638 was observed in cells treated with 5-Aza (70). It is likely that high concentrations of the hypomethylating agent are needed to decrease methylation of the hsa-miR-638 promoter region or that more complex epigenetic mechanisms are regulating this locus (70). Ectopic expression of hsa-miR-203 in cervical cancer cell lines decreased their rates of proliferation and anchorage-independent growth (70). The results described by Wilting *et al* (70) indicate that decreased expression of hsa-miR-149, hsa-miR-203 and hsa-miR-375 in cervical cancer cell lines is associated with the methylation status of their promoter regions.

To investigate the stage in the HR-HPV-mediated transformation process at which hsa-miR-124 becomes methylated, Wilting *et al* (2) analyzed the three loci encoding the mature form of hsa-miR-124 (hsa-miR-124-1/-2/-3) using a longitudinal panel of human foreskin keratinocytes immortalized with HPV 16 and HPV 18. This panel represented the morphological, genetic and epigenetic aspects of the different transformation stages that are observed in high-grade lesions. These authors found that in the late passages of keratinocytes immortalized with HR-HPV, there was an increase in the methylation levels of the loci encoding hsa-miR-124. Furthermore, this increase was correlated with a decrease in miRNA expression and high levels of IGFBP7, which is considered a potential target gene of hsa-miR-124. Wilting *et al* (70) reported that methylation of the promoter regions of hsa-miR-149, hsa-miR-203, hsa-miR-375 and hsa-miR-638 was increased in keratinocytes immortalized with HPV 16 and HPV 18 compared with primary keratinocytes. Moreover, these authors found that the increase in hsa-miR-149, hsa-miR-203 and hsa-miR-375 methylation correlated with malignant progression and that expression of these miRNAs can be restored by treatment with 5-Aza.

The findings in samples from patients with precursor lesions and cervical cancer support the *in vitro* findings. Wilting *et al* (2) reported that no methylation of hsa-miR-124-1 and hsa-miR-124-2 was found in normal cervical tissue. However, in cervical cancer, 90% of samples showed methylation of these genes. Furthermore, these authors observed that the increase in methylation levels of hsa-miR-124-1 and hsa-miR-124-2 was correlated with reduced expression of hsa-miR-124 (2). However, the authors also found that the methylation status of hsa-miR-124-1 and hsa-miR-124-2 was predictive of high-grade lesions in 43 cervical shave biopsy samples from women who tested positive for HR-HPV. They concluded that silencing of hsa-miR-124 by DNA methylation is functionally implicated in cervical carcinogenesis and can be used as a valuable indicator to improve the timely detection of cervical cancer and high-grade precursor lesions. Botezatu *et al* (20) assessed the methylation status of CpG islands surrounding the hsa-miR-124a, hsa-miR-34b and hsa-miR-203 genes in cervical cancer and precursor lesions. They further evaluated the relationship between methylation status and the presence of HPV DNA and the viral genotype. They found significant differences in the methylation levels of the miRNA promoter regions in cervical tumor samples compared with control samples. Hypermethylation of *miR-124a* and *miR-203* was also observed in precursor lesion samples compared with control samples. Botezatu *et al* (20) further observed significant differences in the methylation levels of the miR-124a and miR-203 CpG islands between the HR-HPV group and the low-risk HPV (LR-HPV) group and found a strong association between the methylation process and HR-HPV genotypes. In cervical tumor and precursor lesion samples with LR-HPV genotypes, methylation levels were similar to the ones found in normal samples. This finding strengthens the possibility that the HR-HPV genotypes are involved in miRNA methylation processes (20). Yao *et al* (69) reported that in primary cervical tumors compared with normal tissue, the expression levels of hsa-miR-432, hsa-miR-1286, hsa-miR-641, hsa-miR-1290, hsa-miR-128 and hsa-miR-95 were inversely correlated with the methylation status found in cervical cancer cell lines treated with 5-Aza. Wilting *et al* (2) reported significantly increased methylation levels for hsa-miR-149, hsa-miR-203 and hsa-miR-375 in patients with cervical carcinomas. In high-grade lesions, methylation levels were only significantly increased for hsa-miR-203 and hsa-miR-375. Moreover, Wilting *et al* (70) found an increase in the methylation levels of hsa-miR-203 in cervical samples from women with high-grade lesions who were infected with HPV compared with control samples.

## 8. Conclusion

The finding that alterations in miRNA expression and methylation of key genes involved in cell cycle regulation are frequent events in the process of carcinogenesis represents a challenge and an incentive for this field of research. Extensive study has been devoted to identifying variations in miRNA expression and the expression of miRNA targets in cervical cancer and its precursor lesions. However, knowledge concerning the role of miRNAs in the carcinogenic process is still in its early stages. Evidence suggests that in cervical cancer, hypermethylation of miRNA promoters contributes to the decreased expression of miRNAs with tumor-suppressor gene functions and favors overexpression of miRNAs with oncogenic functions. Methylation is an important mechanism in the HPV viral cycle. Alterations to the methylation status of cellular DNA are influenced by HPV infection, the viral genotype, the physical state of the viral DNA, and oncogenic risk. The E6 and E7 oncoproteins of HPV 16 induce the overexpression of DNA methyltransferase enzymes, which can catalyze the aberrant methylation of protein-coding and miRNA genes that are susceptible to regulation by methylation. Furthermore, HPV deregulates the expression of miRNAs with loci located at fragile sites through the E6 and E7 oncoproteins. Targets of these proteins include transcription factors of miRNAs, such as p53.

## Figures and Tables

**Table I:** Hypermethylated genes associated with cancer development and the biological processes altered during carcinogenesis.

Hypermethylated genes in cancer	Biological process
*hMLH1*, *WRN*, *BRCA1*, ***MGMT***	DNA repair
*CRBP1*, ***RAR-β2***	Vitamin response
*NOREIA*, ***RASSF1A***	Ras signaling
*p15INK4b*, *Rb*, ***P16INK4a***, ***CCNA1***, ***FHIT***	Cell cycle
*P14ARF*, ***p73***, ***HIC-1***	p53 pathway
*E-cadherin*, *H-cadherin*, *FAT*, *EXT-1*, *SLIT2*, *EMP3*, ***CADM1***	Cell adherence and invasion
*TMS1*, *WIF-1*, *SFRP1*, ***hTERT***, ***DcR1***, ***DcR2***, ***DAPK1***	Apoptosis
*DKK-1*, *IGFBP-3*, ***APC***	Wnt signaling pathway
*SOCS.1*, *SOCS-3*, ***SYK***	Tyrosine kinase signaling cascade
*GATA-4*, *GATA-5*, *ID4*	Transcription factors
*GSTP1*, *LKB1/STK11*, *THBS-14*, *COX-2*, *SRBC*, *RIZ1*, *SLC5* *A8*, *TPEF/HPP1*, *Laminin*, ***PTEN***, ***CDH1***, ***TSLC1***	Other pathways

Bold font, promoters reported to be hypermethylated in cervical cancer.

**Table II:** Expression of miRNAs in cervical and uterine cancer.

Study groups	miRNA expression	Significance in cervical cancer	Refs.
Cervical tissue and serum from patients with SCCC with LNM and without LNM and samples from healthy patients	Upregulated: miR-1246, miR-20a, miR-2392, miR-3147, miR-3162-5p and miR-4484	Overexpression of miRNAs in serum can predict gangliar metastasis in patients with early-stage SCCC.	(60)
Samples from patients with primary CAC and SCCC	Upregulated: miR-21, miR-27a, miR-34a, miR-155, miR-196a, miR-203 and miR-221	Differential expression of miRNAs correlates with the histopathological diagnosis of primary CAC and SCCC, independently of clinical stage and HPV infection.	(61)
Samples from patients with HSIL and CAC and samples from healthy patients	Upregulated: hsa-miR-9, hsa-miR-15b and hsa-miR-28-5p Downregulated: hsa-miR-100 and hsa-miR-125b	Altered expression of these five miRNAs, is associated with chromosomal alterations in cervical cancer.	(62)
Samples from patients with SCCC (FIGO IB2-IV) and patients with early-stage SCCC (FIGO IB1)	Downregulated: hsa-let-7c, hsa-miR-10b, hsa-miR-100, hsa-miR-125b, hsa-miR-143, hsa-miR-145 and hsa-miR-199a-5p	Decreased expression of let-7c, miR-10b, miR-100, miR-125b, miR-143, miR-145 and miR-199a-5p is associated with advanced-stage SCCC. Decreased expression of let-7c, miR-100, miR-125b, miR-143, miR-145 and miR-199a-5p is associated with LNM and decreased patient survival. Decreased expression of miR-10b and miR-100 is associated with a poor prognosis for SCCC.	(63)
Samples from patients with SCCC (CIN2, CIN3) and samples from healthy patients	Upregulated: miR-518a, miR-34b, miR-34c, miR-20b, miR-338, miR-9, miR-512-5p, miR-424, miR-345 and miR-10a Downregulated: miR-193b and miR-203	Differential miRNA expression was found in tissues from patients with SCCC and samples from healthy patients. Predictive target analysis revealed that the miRNAs with decreased expression control signaling pathways regulating cell cycle and apoptosis.	(64)
Samples from patients with cervical cancer (IB–IIB) and patients with benign gynecological diseases	Upregulated: hsa-miR-15a, hsa-miR-19a, hsa-miR-20b, hsa-miR-21, hsa-miR-141, hsa-miR-106b and miR-hsa-224 Downregulated: hsa-let-7c, hsa-miR-143, hsa-miR-199a-5p, hsa-miR-203 and miR-145	hsa-miR-15a, hsa-miR-106b, and hsa-miR-20b regulate a large number of target genes and have strong regulatory effects on the differential expression of genes in cervical cancer.	(65)
Samples from patients with cervical cancer, LSIL, HSIL and healthy patients	Upregulated: miR-522*, miR-512-3p, miR-148a, miR-302b, miR-10a, miR-196a and miR-132 Downregulated: miR-26a, miR-143, miR-145, miR-99a, miR-203, miR-513, miR-29a, miR-199a, miR-106a, miR-205, miR-197, miR-16, miR-27a and miR-142-5p	Different miRNA expression was found between normal cervix, precursor lesions, and cancer tissues. This suggests that deregulated miRNAs play a role in malignant transformation of cervical cells.	(66)
Samples from patients with cervical cancer	Upregulated: miR-21, miR-200a and miR-9 Downregulated: miR-203 and miR-218	miR-200a affects the metastatic potential of cervical cancer cells by suppressing the expression of genes that are important for cell motility	(67)
Samples from patients with cervical cancer and healthy patients	Upregulated: miR-15b, miR-16, miR-146a, miR-155, miR-223, miR-21, miR-205 and let-7f Downregulated: miR-126, miR-424, miR-143, miR-145	Functional studies showed that miR-143 and miR-145 suppress cell growth, whereas miR-146a promotes cell proliferation in cervical cancer.	(1)
Cervical cancer cell lines CaSki, SiHa, HeLa and C33A. Samples from patients with cervical cancer and healthy patients	Upregulated: miR-182, miR-183 and miR-210 Downregulated: miR-143, miR-145, miR-126, miR-195, miR-218, miR-368 and miR-497	miR-218 expression was decreased in HPV-positive cell lines and cervical cancer tissue compared with C33A cells and normal cervix tissue. Expression of the HPV 16 (high-risk) E6 oncoprotein decreases the expression of miR-218 compared with HPV 6 (low-risk). This suggests that some miRNAs are regulated by HPV.	(11)
Samples from patients with invasive SCCC and healthy patients	Upregulated: miR-199a, miR-199s, miR-9, miR-199a, miR-199b, miR-145, miR-133a, miR-133b, miR-214 and miR-127 Downregulated: miR-149 and miR-203	Overexpression of miR127 is associated with LNM. *In vitro*, transfection with anti-miR-199a in cervical cancer cell lines inhibits cell growth.	(68)
Cervical cancer cell lines SW756, C4I, C33A, CaSki, SiHa and ME-180 as well as samples from patients with benign gynecological pathologies	Upregulated: miR-21 Downregulated: let-7b, let-7c, miR-23b, miR-196b and miR-143	Decreased expression of miR-143 and overexpression of miR-21 in cervical cancer samples is reproducible, which highlights the potential value of miRNAs as tumor markers.	(5)

SCCC, squamous cervical cell carcinoma; CAC, cervical adenocarcinoma; HSIL, high-grade squamous intraepithelial lesion; LSIL, low-grade squamous intraepithelial lesion; LNM, lymph node metastasis; FIGO, classification criteria of the International Federation of Gynecology and Obstetrics.

**Table III:** miRNA genes regulated by methylation in certain types of cancer.

Type of cancer	Methylation status	Refs.
Colon cancer	Hypermethylated: miR-126, miR-34a, miR-34b/c, miR-1-1, miR-133a-2 and miR-149	(60,71–73)
Gastric cancer	Hypermethylated: miR-433, miR-127, miR-148a, miR-34b, miR-129, miR-9, miR-10b, miR-195 and miR-378	(74–79)
Leukemia	Hypermethylated: miR-663	(80)
Bladder cancer	Hypermethylated: miR-200b, miR-152 and miR-10a	(81)
Hepatocellular carcinoma	Hypermethylated: miR-129-2, miR-10a, miR-122 and miR-1-1	(19,50,82,83)
	Hypomethylated: miR-191	(84)
Breast cancer	Hypermethylated: miR-31, miR-130a, let-7a-3/let-7b, miR-155, miR-137, miR-34b/miR-34c, miR-125b and miR-34a	(72,85–87)
Prostate cancer	Hypermethylated: miR-205, miR-132 and miR-193b	(88–90)
Non-small cell lung cancer	Hypermethylated: miR-9-3, miR-122, miR-124-2, miR-124-3 and miR-34b/c	(91–94)
Multiple myeloma	Hypermethylated: miR-203	(95)
Pancreatic cancer	Hypermethylated: miR-132	(85)
	Hypomethylated: miR-200a and miR-200b	(14)
Ovarian cancer	Hypermethylated: miR-34a and miR-34b/c	(72)
